# Body composition, adipokines, bone mineral density and bone remodeling markers in relation to IGF-1 levels in adults with Prader-Willi syndrome

**DOI:** 10.1186/s13633-018-0055-4

**Published:** 2018-01-16

**Authors:** I. Caroline van Nieuwpoort, Jos W. R. Twisk, Leopold M. G. Curfs, Paul Lips, Madeleine L. Drent

**Affiliations:** 1 Department of Internal Medicine, Section Endocrinology, VU University Medical Center and Amsterdam Neuroscience, De Boelelaan 1117, 1081 HV Amsterdam, The Netherlands; 2 Department of Clinical Epidemiology and Biostatistics and EMGO Institute, VU University Medical Center and Institute of Health Sciences, VU University, Amsterdam, The Netherlands; 3 Department of Clinical Genetics, Maastricht University Medical Center, Maastricht, The Netherlands

**Keywords:** PWS, IGF-1, Body composition, Appetite regulating peptides, Bone mineral density

## Abstract

**Background:**

In patients with Prader-Willi syndrome (PWS) body composition is abnormal and alterations in appetite regulating factors, bone mineral density and insulin-like growth factor-1 (IGF-1) levels have been described. Studies in PWS adults are limited. In this study, we investigated body composition, appetite regulating peptides, bone mineral density and markers of bone remodeling in an adult PWS population. Furthermore, we investigated the association between these different parameters and IGF-1 levels because of the described similarities with growth hormone deficient patients.

**Methods:**

In this cross-sectional observational cohort study in a university hospital setting we studied fifteen adult PWS patients. Anthropometric and metabolic parameters, IGF-1 levels, bone mineral density and bone metabolism were evaluated. The homeostasis model assessment of insulin resistance (HOMA2-IR) was calculated. Fourteen healthy siblings served as a control group for part of the measurements.

**Results:**

In the adult PWS patients, height, fat free mass, IGF-1 and bone mineral content were significantly lower when compared to controls; body mass index (BMI), waist, waist-to-hip ratio and fat mass were higher. There was a high prevalence of osteopenia and osteoporosis in the PWS patients. Also, appetite regulating peptides and bone remodelling markers were aberrant when compared to reference values. Measurements of body composition were significantly correlated to appetite regulating peptides and high-sensitive C-reactive protein (hs-CRP), furthermore HOMA was correlated to BMI and adipokines.

**Conclusion:**

In adults with Prader-Willi syndrome alterations in body composition, adipokines, hs-CRP and bone mineral density were demonstrated but these were not associated with IGF-1 levels. Further investigations are warranted to gain more insight into the exact pathophysiology and the role of these alterations in the metabolic and cardiovascular complications seen in PWS, so these complications can be prevented or treated as early as possible.

## Background

Prader-Willi syndrome (PWS), first described in 1956, is a genetic disorder caused by abnormalities on chromosome 15. Hypothalamic dysfunction is supposed to play a causal role in the characteristic clinical symptoms such as feeding difficulties, hypotonia, hyperphagia, morbid obesity, endocrine dysfunction, short stature, behavioral abnormalities, mental retardation, high pain threshold and alterations in temperature regulation [1–5]. Most patient with PWS are (morbidly) obese and their body composition (BC) is aberrant with a decreased muscle and lean body mass. Not only in obese but also in non-obese PWS patients fat mass is increased in comparison to subjects with simple obesity and a comparable body mass index (BMI) [6–12]. The abnormalities in body composition described in PWS are very comparable to those seen in patients with growth hormone deficiency (GHD).

Since hypothalamic dysfunction might be an important causal factor in PWS and hunger and satiety are regulated by the hypothalamus, a number of studies investigated concentrations of appetite regulating peptides in children and adults with PWS. The exact underlying mechanisms of the physiology of appetite regulating hormones or adipokines and their possible pathophysiological role in the development of obesity and metabolic and cardiovascular diseases are not yet fully understood, especially not in PWS patients. It is known that ghrelin, mainly secreted from the stomach, is an orexigenic peptide which stimulates food intake and is decreased in normal subjects after a meal [13]. Plasma ghrelin levels are elevated in PWS patients when compared to healthy or obese controls, possibly even at young age before the development of obesity [5, 14–20]. Leptin is an appetite-suppressing peptide which also promotes energy expenditure and is secreted predominantly by adipose tissue [13, 21]. Leptin levels are positively correlated to BMI and are known to be elevated in obese subjects, also known as “leptin resistance”. After fasting or weight loss, leptin levels decrease. However, leptin was initially described as having a pro-inflammatory function [21]. Data on leptin concentrations in PWS patients are conflicting, showing normal or elevated levels [5, 22–27]. Adiponectin, secreted by adipocytes, is known to have anti-atherogenic and anti-inflammatory effects and also has effects on lipid and glucose metabolism as it improves insulin sensitivity and glucose uptake [5, 13, 21]. Fasting adiponectin levels are reported to be higher in PWS subjects when compared to controls with simple obesity, but lower or normal when compared to lean control subjects [5, 11, 26, 28, 29]. Levels of resistin, secreted by macrophages and white adipose tissue, are elevated in human subjects with obesity. Although resistin causes insulin resistance in rodents, in humans it seems to have a more pro-inflammatory effect than a role in glucose homeostasis [21]. Studies on levels of resistin in PWS subject are scarce, but a significantly higher resistin concentration was reported in PWS patients in comparison with both healthy lean subjects and subjects with obesity [26].

Since PWS is characterized by obesity with a high fat percentage, one could expect an unfavorable glucose homeostasis and lipid profile with an increased risk for cardiovascular disease. However, data on metabolic profiles have been conflicting, with studies showing elevated plasma insulin levels indicating insulin resistance, but also normal or even increased insulin sensitivity in comparison to subjects with simple obesity has been reported [10, 12, 30–33]. Lipid profile seems to be within the normal range or comparable to subjects with simple obesity [11, 12, 23, 31]. The concentration of high-sensitive C-reactive protein (hs-CRP), as a marker of low-grade systemic inflammation and known predictor of cardiovascular disease, is reported to be elevated in subjects with PWS [34–36]. Studies with regard to bone mineral density (BMD) in PWS patients show that BMD is decreased and markers of bone turnover are elevated [23, 37, 38].

The aim of the present study was to investigate body composition, appetite regulating peptides, BMD and markers of bone remodeling in an adult population of PWS patients. Healthy brothers and sisters of the PWS patients served as a control group for part of these measurements. Furthermore, we analyzed IGF-1 levels because of the described similarities of PWS patients with GHD patients. We hypothesized that that body composition, appetite regulating peptides, BMD and markers of bone remodeling are deviant in the PWS patients and expected to find an association with IGF-1 levels, at least for part of these parameters. We also investigated whether there was a gender difference within the PWS patients.

## Subjects and methods

### Subjects

Subjects for this study were recruited via the Dutch Prader-Willi patients’ association, as described before [39]. In summary, we included fifteen PWS patients with genetically confirmed PWS, eleven females and four males, median age 22.0 years (range 19.2–42.9). Patients were excluded if they had received GH-treatment within three months before enrolment. Written informed consent was obtained from the patients and their parents or caretakers. The study was approved by the Medical Ethics Review Committee of the VU University Medical Center and was conducted according to the principles of the Helsinki Declaration.

### Control group

Fourteen healthy brothers and sisters of the included PWS patients constituted the control group, seven females and seven males, median age 28.5 years (range 17.5–41.3). They all had a good general health without a history of pituitary disease, surgery or radiotherapy of the head. IGF-1 levels were within the normal range for age and gender in all control subjects.

### Methods

Medical history was taken in all PWS patients and subjects in the control group and a general physical examination was performed. In all participants, height (m), weight (kg), waist and hip circumference (cm) were measured and BMI (kg/m^2^) and waist-to-hip ratio (WHR) were calculated as described earlier [39].

### Genetic analysis

In all patients, the diagnosis of PWS was genetically confirmed as described before at the Department of Clinical Genetics, Maastricht University Medical Center, The Netherlands [39].

### Laboratory tests

Main laboratory tests were performed in PWS patients only. Blood samples were drawn at approximately 08:00–10:00 am after an overnight fast. Standard laboratory tests were done to rule out any underlying disease and revealed no relevant abnormalities. Blood samples for hormonal assessments were drawn at the same time, but were analyzed altogether at the end of the study. In healthy controls only IGF-1 measurements were performed. IGF-1 and IGFBP-3 (insulin-like growth factor binding protein-3): Immunometric assay, Luminescence, Immulite 2500® laboratory assay, Siemens Medical Solutions Diagnostics, USA. Reference range gender- and age-specific. Both IGF-1 concentration and IGF-1 standard deviation scores (SDS) were used in the analysis. 25-Hydroxy-vitamin D: Competitive binding protein assay, after extraction, Diasorin, Stillwater Minnesota, USA. Lower limit of quantitation: 5 nmol/L. Mean intra-assay % CV at level of 8 nmol/L is 12%, at level of 25 nmol/L mean intra-assay % CV is 9% and at level of 100 nmol/L mean intra-assay % CV is 7%. Reference values: 25–125 nmol/L. Osteocalcin: Immunometric assay (colorimetric), BioSource, Nivelles, Belgium. Lower limit of quantitation: 0.4 nmol/L. Mean intra-assay % CV < 5% at whole range. Reference values: 0.4–4.0 nmol/L. Insulin: Immunometric assay, Luminescence, Advia Centaur, Siemens Medical Solutions Diagnostics, USA. Lower limit of quantitation: 10 pmol/L. Mean intra-assay % CV at level of 20 pmol/L is 4%, at level of 500 pmol/L mean intra-assay % CV is 3% and at level of 1500 pmol/L mean intra-assay % CV is 4%. Reference values (fasting): 12–96 pmol/L. Hs-CRP: Immunometric assay (colorimetric), in-house with rabbit anti-CRP antibodies from DAKO. Lower limit of quantitation: 0.1 mg/L. Mean intra-assay % CV at level of 0.2 mg/L is 4%, at level of 2 mg/L mean intra-assay % CV is 5% and at level of 7 mg/L mean intra-assay % CV is 6%. Reference values: < 1.0 low risk cardiovascular disease, 1.0–3.0 intermediate risk, > 3.0 high risk. Adiponectin: Radioimmunoassay, Linco Research Inc., St. Charles Missouri USA. Lower limit of quantitation: 0.5 mg/L. Mean intra-assay % CV is 5% over the whole range. Reference values: 0.8–48 mg/L. Ghrelin: Radioimmunoassay, Linco Research Inc., St. Charles Missouri, USA. Lower limit of quantitation: 240 ng/L. Mean intra-assay % CV is 4% over the whole range. Leptin: Radioimmunoassay, Linco Research Inc., St. Charles, Missouri, USA. Lower limit of quantitation: 0.5 μg/L. Mean intra-assay % CV at level of 5 μg/L is 8% and at level of 25 μg/l mean intra-assay % CV is 3%. Reference values for men (BMI 18–25) 2.5–8 μg/L, women (BMI 18–25) 4.5–16 μg/L. Resistin: Immunometric assay (colorimetric), Biovendor Laboratory Medicine, INC, Modrice, Czech Republic. Lower limit of quantitation: 0.8 ng/ml. Mean intra-assay % CV is 5% over the whole range. Reference values: 4.1–12.1 ng/ml. All other hormonal/laboratory assessments were performed at the endocrine laboratory of the VU University Medical Center with commercially available, regularly internal and external quality controlled immunoassays. The homeostasis model assessment of insulin resistance (HOMA2-IR) was calculated with fasting glucose and insulin levels by the HOMA2 calculator as provided by the website of the University of Oxford (http://www.dtu.ox.ac.uk/homacalculator).

### Urine analysis

A fasting 2-h urine sample was used for measurements of creatinine, calcium and hydroxyproline in PWS patients. Reference values for creatinine: 5.3–17.7 mmol/24 h, for calcium: 2.5–8.0 mmol/24 h, for hydroxyproline: < 25 mmol/mol creatinine.

### Bioelectric impedance analysis (BIA)

In both PWS patients and healthy controls body composition was measured by BIA using RJL 101/S/D; Akern-Rijl Systems. For BIA calculations, the method as described by Lukaski et al. [40] was used.

### Dual-energy X-ray absorptiometry (DXA)

In PWS subjects only, body composition (BC) and bone mineral density (BMD) were measured using the Hologic QDR-4500. BMD was measured at the lumbar spine at the L1-L4 level and at the hip region (total hip).

### Statistical analysis

All analyses were performed using the statistical software package SPSS version 21 (SPSS, Chicago, IL, USA). All data are presented as median with interquartile range (IQR) unless stated otherwise since most variables were not normally distributed. For comparison between patients and controls and between males and females the Mann-Whitney U test was used. Spearman’s rank correlation test was used to analyze correlations between pairs of variables. A *p*-value of < 0.05 was considered statistically significant and all test were two-tailed.

## Results

Fifteen adult PWS patients were included in this study. Two subjects used a vitamin D supplement, two persons a multivitamin supplement. One subject was treated with a bisphosphonate. Four of the PWS patients were treated with GH in the past (treatment duration 7–12 years), but all stopped treatment at least 5 months before the study. Two of the patients suffered from hypothyroidism and were treated with thyroxin in a stable dose, one of the patients used hydrocortisone in a stable dose because of adrenal insufficiency. Only two of the fifteen patients (13%) had normal gonadal function, as was published earlier [39]. Of the hypogonadal patients, 54% was treated with sex steroids. None of the subjects received treatment for diabetes mellitus or was treated with statins. In three subjects, BMD of the lumbar spine could not be measured because of the presence of osteosynthesis material, furthermore one subject refused measurement of total body BMD.

Table 1 shows body composition characteristics of the PWS patients and controls, as well as measurements of bone mineral density in the PWS patients. In the whole group of PWS patients, height, IGF-1, fat free mass (FFM) and bone mineral content (BMC) measured by BIA were significantly lower when compared to controls; BMI, waist, WHR and fat mass (FM) were significantly higher. In the PWS subjects, T- and Z-scores of the of BMD of the lumbar spine and total hip were low. Two patients met the criteria for osteoporosis of the lumbar spine, six the criteria for osteopenia; at the total hip level, two met the criteria for osteoporosis and ten had osteopenia. Although there were only four males included in the PWS group, we found significantly lower T-scores of the lumbar spine and hip in men when compared to women. As expected, we found a significant higher height, weight, WHR and FFM in the males than in the females of the control group. In the PWS group these sex differences were not seen, however as mentioned before there were only four males included. We were not able to analyze differences between genetic subtypes since fourteen PWS patients had a paternal deletion and only one subject a maternal uniparental disomy.

**Table 1:** Body composition characteristics and measurements of bone mineral density in PWS patients and control group

	PWS						Controls					
	Total group		Men		Women		Total group		Men		Women	
	(*N* = 15)	IQR	(*N* = 4)	IQR	(*N* = 11)	IQR	(*N* = 14)	IQR	(*N* = 7)	IQR	(*N* = 7)	IQR
Age, years	22.0	12.9	26.6	18.3	20.9	12.9	28.5	18.0	28.4	17.4	28.7	20.1
Sex												
Female, %	73.3						50.0					
Male, %	26.7						50.0					
Genetic subtype												
Deletion, %	93.3											
UPD, %	6.7											
Height, m	1.58**	0.08	1.60	0.09	1.57	0.10	1.77	0.17	1.86##	0.066	1.69	0.048
Weight, kg	69.5	33.6	67.9	32.9	70.3	37.1	73.1	15.9	79.1#	15.9	65.2	14.1
BMI, kg/m^2^	27.5*	16.7	26.1	13.2	32.1	20.3	22.4	4.6	22.7	5.1	22.0	5.8
Waist, cm	94.8*	27.5	94.3	17.1	94.8	43.8	80.2	19.3	84.5	11.4	71.2	24.2
Hip, cm	107	33.3	102.5	21.1	120.2	34.2	98.9	8.0	102.1	5.4	97.8	15.3
WHR	0.87*	0.13	0.93	0.12	0.87	0.10	0.82	0.14	0.84#	0.07	0.73	0.10
FM, kg (BIA)	28.5**	23.5	25.4	21.6	30.1	23.5	14.2	9.9	13.8	2.9	14.5	10.0
FFM, kg (BIA)	44.2**	16.2	44.2	16.1	44.2	16.2	56.2	18.0	66.2#	13.6	48.5	5.7
FM, % (BIA)	42.4**	12.4	37.5	12.3	42.4	11.1	20.6	12.3	16.7	4.8	25.1	12.1
FFM, % (BIA)	57.6**	12.4	62.5	12.4	57.6	11.1	79.4	12.3	83.3	4.8	74.9	12.1
BMC, % (BIA)	30.0**	11.4	32.5	8.1	29.7	12.2	42.8	9.7	44.5	7.3	40.3	22.1
BMC Lumbar spine, g (DXA)	41.0	13.8	41.7	17.0	41.0	11.4						
BMD Lumbar spine, g/cm^2^ (DXA)	0.91	0.17	0.80	0.28	0.93	0.20						
T-score Lumbar spine, SDS (DXA)	−1.4	2.0	−2.6#	2.5	−0.9	1.5						
Z-score Lumbar spine, SDS (DXA)	−1.7	1.4	−2.6	2.4	− 1.0	1.7						
BMC Total hip, g (DXA)	23.0	5.1	22.9	3.5	23.0	8.4						
BMD Total hip, g/cm^2^ (DXA)	0.76	0.10	0.74	0.13	0.76	0.15						
T-score Total hip, SDS (DXA)	−1.6	0.9	−2.0#	0.9	−1.2	1.5						
Z-score Total hip, SDS (DXA)	−1.5	1.1	−2.0	0.8	−1.4	1.3						
Total BMC, g (DXA)	1763	386	1684	368	1763	423						
Total BMD, g/cm^2^ (DXA)	0.99	0.13	0.95	0.14	0.99	0.14						
FM, kg (DXA)	33.5	19.4	28.5	17.1	39.5	26.5						
LBM, kg (DXA)	40.5	13.0	41.3	13.1	40.5	15.3						
FM, % (DXA)	45.1	11.4	40.4	9.3	47.8	12.5						

*BMI* body mass index, *WHR* waist-to-hip ratio, *FM* fat mass, *FFM* fat free mass, *BMC* bone mineral content, *BMD* bone mineral density, *LBM* lean body mass

All data are presented as median with interquartile range (IQR)

* Significant difference when compared to control group, *p* < 0.05; ** *p* < 0.01

# Significant difference when compared to other sex within group, *p* < 0.05; ## *p* < 0.01

In Table 2 (panel A), markers of metabolic status, bone remodeling markers and appetite regulating factors in PWS patients are displayed, as well as IGF-1 levels in PWS patients (panel A) and healthy controls (panel B). There were no significant differences between male and female PWS adults. One patient had an HbA1c level which was elevated and treatment for diabetes mellitus was started. Only three PWS patients had a hs-CRP concentration < 3 mg/L. Adiponectin levels were within reference values in all subjects. Three of the four male and nine of the eleven female PWS subjects had leptin levels above reference values. Resistin levels were below the reference value in seven of the fifteen PWS subjects and normal in the remaining subjects. IGF-1 concentration and IGF-1 SDS were significantly lower in PWS subjects when compared to healthy controls. Although mostly within normal range, osteocalcin levels were low in PWS patients.

**Table 2:** IGF-1 levels, markers of metabolic status, bone remodeling markers and appetite regulating factors in PWS patients (panel A) and IGF-1 levels in healthy controls (panel B)

Panel A	PWS					
	Total group		Men		Women	
	(*N* = 15)	IQR	(*N* = 4)	IQR	(*N* = 11)	IQR
IGF-1, nmol/L	14.6	7.7	16.0	11.9	14.6	7.7
IGF-1, SDS	−1.94	1.24	−1.55	1.24	−1.96	1.00
IGFBP-3, mg/L	4.30	1.38	4.73	2.87	4.02	1.89
Calcium, mmol/L	2.42	0.17	2.43	0.30	2.42	0.09
Albumin, g/L	43.0	5.0	42.5	12.0	43.0	4.0
Creatinine, μmol/L	69.0	10.0	71.0	28.0	69.0	10.0
Alkaline phosphatase, U/L	103.0	54.0	98.0	57.0	106.0	61.0
Phosphate, mmol/L	1.12	0.31	0.91	0.47	1.16	0.26
25(OH)D, nmol/L	61.0	34.0	83.5	66.0	54.0	31.0
Osteocalcin, nmol/L	0.90	2.49	0.77	2.28	1.24	2.49
Total cholesterol, mmol/L	4.8	1.8	4.9	2.6	4.8	1.8
HDL, mmol/L	1.3	0.5	1.2	0.7	1.3	0.4
LDL, mmol/L	2.9	1.2	2.1	NA	3.2	1.0
Triglycerides, mmol/L	0.80	0.30	1.75	2.60	0.80	0.30
Glucose, mmol/L	4.2	1.0	4.2	0.2	4.0	1.3
HbA1c, %	5.4	0.4	5.6	3.1	5.4	0.4
Insulin, pmol/L	30.6	36.8	37.2	179.1	29.1	38.5
HOMA index	0.58	0.62	0.66	3.04	0.55	0.64
Leptin, μg/L	21.5	33.2	20.1	38.7	24.9	33.2
Ghrelin, ng/L	2476	2080	2118	2201	2612	2092
Resistin, ng/ml	4.4	3.6	4.3	5.7	4.4	3.6
Adiponectin, mg/L	13.9	6.7	13.2	9.1	13.9	6.4
Hs-CRP, mg/L	4.04	10.39	3.48	8.01	7.54	19.68
Urine Calcium, mmol/24 h	0.20	0.5	0.55	1.1	0.20	0.2
Urine Creatitine, mmol/24 h	1.74	1.47	3.27	2.86	1.70	0.69
Urine Hydroxyprolin, mmol/24 h	0.046	0.028	0.055	0.087	0.046	0.027
Urine Hydroxyprolin/Creatinin ratio	25.2	21.2	23.2	40.9	25.2	20.4
Panel B	Controls					
	Total group		Men		Women	
	(*N* = 14)	IQR	(*N* = 7)	IQR	(*N* = 7)	IQR
IGF-1, nmol/L	21.8	11.6	21.4	14.2	22.1	10.5
IGF-1, SDS	−0.79	1.06	−1.00	1.07	− 0.76	1.72

All data are presented as median with interquartile range (IQR)

In Table 3, correlation coefficients are shown between measurements of body composition, IGF-1, appetite regulating peptides and measurements of glucose homeostasis within the total group of PWS patients. Measurements of body composition as measured by BIA correlated significantly with those measured by DXA (data not shown), we therefore only display the correlation coefficients for FM and lean body mass (LBM) as measured by DXA for a better overview. We found a significant correlation between IGF-1 concentration and age (r − 0.54, p 0.04) and IGF-1 concentration and IGFBP-3 (r 0.70, p 0.004) in the total PWS group (data not shown). There were no significant correlations between IGF-1, IGFBP-3 or IGF-1/IGFBP-3 ratio and measurements of body composition, HOMA or adipokines. There was a significant positive correlation between BMI and FM, LBM, HOMA index, leptin, resistin and hs-CRP. Also, there was a significant negative correlation between HbA1c and adiponectin and between HOMA index and ghrelin. Leptin had a significant positive correlation with BMI, FM, LBM, glucose, insulin, HOMA index and hs-CRP. Furthermore, there was a significant negative correlation between adiponectin and resistin. There was no significant correlation between age and appetite regulating peptides. We also investigated whether there were significant correlations between IGF-1 and measurements of BMC, BMD and markers of bone metabolism. We found a significant correlation between IGF-1 SDS and alkaline phosphatase (r − 0.624, *p* < 0.05). Correlations between IGF-1 concentration and alkaline phosphatase and between IGF-1 SDS and BMC of the lumbar spine were nearly significant. There was a positive, but non-significant, correlation between osteocalcin and IGF-1, IGFBP-3 and IGF-1/IGFBP-3 ratio (data not shown). Because of a lack of power, we were not able to investigate differences in correlation coefficients between male and female subjects or between genetic subtypes within the PWS group.

**Table 3:** Correlation coefficients of measurements of body composition, IGF-1, appetite regulating peptides and measurements of glucose homeostasis within the PWS patients (total group)

	BMI	FM	LBM	IGF-1	Glucose	HbA1c	Insulin	HOMA	Leptin	Ghrelin	Resistin	Adiponectin	Hs-CRP
BMI (kg/m^2^)	1.00	0.96**	0.75**	− 0.18	0.19	0.16	0.44	0.55*	0.83**	−0.44	0.58*	−0.31	0.77**
FM (g, DXA)	0.96**	1.00	0.69**	−0.14	0.19	0.18	0.36	0.46	0.78**	−0.33	0.53*	−0.30	0.88**
LBM (g, DXA)	0.75**	0.69**	1.00	0.02	0.02	0.32	0.46	0.51	0.54*	−0.46	0.69**	−0.44	0.66**
IGF-1 (nmol/L)	−0.18	−0.14	0.02	1.00	−0.03	−0.18	0.26	0.04	−0.04	−0.11	0.01	−0.39	− 0.27
Glucose (mmol/L)	0.19	0.19	0.02	−0.03	1.00	0.14	0.67**	0.65*	0.51*	−0.34	0.20	−0.45	0.39
HbA1c (%)	0.16	0.18	0.32	−0.18	0.14	1.00	0.02	0.09	0.14	0.10	0.35	−0.58*	0.41
Insulin (pmol/L)	0.44	0.36	0.46	0.26	0.67**	0.02	1.00	0.99**	0.69**	−0.48	0.39	−0.46	0.33
HOMA (index)	0.55*	0.46	0.51	0.04	0.65*	0.09	0.99**	1.00	0.67**	−0.58*	0.54*	−0.34	0.41
Leptin (μg/L)	0.83**	0.78**	0.54*	−0.04	0.51*	0.14	0.69**	0.67**	1.00	−0.41	0.37	−0.39	0.66**
Ghrelin (ng/L)	−0.44	−0.33	− 0.46	−0.11	− 0.34	0.10	− 0.48	−0.58*	− 0.41	1.00	− 0.31	0.29	− 0.24
Resistin (ng/ml)	0.58*	0.53*	0.69**	0.01	0.20	0.35	0.39	0.54*	0.37	−0.31	1.00	−0.56*	0.51*
Adiponectin (mg/L)	−0.31	−0.30	− 0.44	−0.39	− 0.45	−0.58*	− 0.46	−0.34	− 0.39	0.29	− 0.56*	1.00	− 0.47
Hs-CRP (mg/L)	0.77**	0.88**	0.66**	−0.27	0.39	0.41	0.33	0.41	0.66**	−0.24	0.51*	−0.47	1.00

* Significant correlation, *p* < 0.05; ** *p* < 0.01

## Discussion

In this study we investigated body composition, bone mineral density, markers of bone remodeling, appetite regulating peptides and IGF-1 levels in a group of PWS adults. As expected, there was a significant difference in body composition between PWS subjects and healthy controls, with a higher fat mass and lower lean body mass in PWS patients. It must be noted that our study group of PWS patients, with a median BMI of 27.5 kg/m^2^, was only mildly obese when compared to previously studied PWS adults. IGF-1 levels were decreased in PWS subjects when compared to healthy controls. Since there was no significant correlation between BMI and both IGF-1 concentration and IGF-1 SDS, this reduced level of IGF-1 is not solely explained by obesity. We found no significant correlations between IGF-1, IGFBP-3 or IGF-1/IGFBP-3 ratio and measurements of body composition, HOMA or adipokines. In an earlier study we have already described the presence of growth hormone deficiency in a part of our study population [39]. GH treatment within three months prior to the study period was an exclusion criterion, but four of the PWS patients were treated with GH in the past as mentioned before. This might have influenced our results since growth hormone has effects on body composition, insulin sensitivity, adipokines and bone mineral density. Also, hypogonadal state might have influenced body composition measurements in our patient group, since 46% of the PWS patients with hypogonadism were not treated with sex hormones.

In agreement with other studies, leptin and hs-CRP concentrations were elevated in the majority of our PWS patients and adiponectin levels were within the reference range in all patients [23, 25–27, 34–36, 41, 42]. Resistin levels were normal or decreased, which is in contrast to earlier reported data in PWS patients and to studies in simple obesity, where resistin levels are known to be elevated [26]. There was a significant positive correlation between BMI and leptin, resistin, HOMA-IR and hs-CRP, and also between HOMA index and leptin and ghrelin. Many of the correlations we found are consistent with earlier published results in PWS patients and subjects with simple obesity.

BMD is reported to be decreased and markers of bone turnover elevated in PWS subjects [23, 37, 38]. In accordance we found a high prevalence of osteopenia and osteoporosis in our PWS subjects, most pronounced in the male subjects. This was accompanied by low levels of osteocalcin, which is produced by osteoblast and used as a marker of bone formation. The abnormal body composition, low levels osteocalcin and higher prevalence of osteopenia and osteoporosis seen in PWS patients could, at least partially, be explained by endocrine disorders such as growth hormone deficiency and hypogonadism. Data on the GH/IGF-1 axis and BMD or bone metabolism in PWS adults are very scarce. We found a near significant positive correlation between IGF-1 SDS and BMC of the lumbar spine and a significant negative correlation between IGF-1 SDS and alkaline phosphatase. There was a positive, but non-significant, correlation between osteocalcin and IGF-1, IGFBP-3 and IGF-1/IGFBP-3 ratio. Concentrations of osteocalcin were low, which was more pronounced in men than in women, although not significantly. This may indicate a low level of bone formation. It is known that IGF-1 is an important stimulatory factor for bone formation. That we did not find a significant correlation between IGF-1 levels and osteocalcin in this study could be a result of the small number of patients included and the low levels of both IGF-1 and osteocalcin with a small spread within the PWS patients. Since there was a significant negative correlation between IGF-1 and alkaline phosphatase in our PWS patients, there may be a disturbance between bone formation and bone resorption in PWS patients, resulting in low BMD, which might be related to alterations in IGF-1 levels. However, both IGF-1 levels and bone remodelling are known to be influenced by sex steroids. In PWS patients there is a high prevalence of hypogonadism [39, 43, 44]. In our total group of PWS subjects, there was a significant negative correlation between testosterone levels and BMD of the lumbar spine (r − 0.674, p 0.016) and a near significant positive correlation between estradiol concentration and BMD of the total hip (r 0.509, p 0.052). These correlations were not significant when analysed for both sexes separately (data not shown), but as mentioned earlier those groups were small. It is possible that the low BMD we found in our study is caused by (a combination of) low IGF-1 levels as well as a hypogonadal state. Furthermore, a lower level of physical activity with less weight-bearing stress on the bones could also attribute to a lower BMD in PWS patients [5]. Median 25-hydroxy-vitamin D level was 61 nmol/L in our PWS study population, therefore vitamin D deficiency as a cause of low BMD is unlikely. We are not informed about fractures in our study population, therefore we could not investigate whether there is a relation between IGF-1 levels, BMD and fracture risk.

There are some limitations to our study. We included a relatively small number of patients and we performed several statistical tests on the study data. Therefore it is difficult to draw hard conclusions from our study results. However, since PWS has a low prevalence, it is very difficult to perform a study like ours in large patient groups. We tested for differences between both sexes and the results indicate that there is a difference for some of the investigated variables, but since there were only four male PWS subjects included, the results should be interpreted with caution. Sex differences in correlation coefficients and differences between genetic subtypes of PWS could not be analyzed. Unfortunately no measurements of appetite regulating hormones and DXA have been performed in the healthy controls. Therefore, it was not possible to investigate correlations between these different parameters in the control subjects or compare the results found in PWS patients to the control group.

Life expectancy in individuals with Prader-Willi syndrome is decreased and this shortened lifespan is mainly the result of metabolic, cardiovascular and respiratory complications. There might be a difference in causes of death between gender and genetic subtype of PWS [45]. Our results indicate that there is a relation between an increased BMI and a higher level of insulin resistance and low-grade systemic inflammation which is likely to attribute to the high percentage of metabolic and cardiovascular morbidity and mortality that has been described in PWS adults. There is no cure for PWS and therefore treatment should be aimed at preventing the complications seen in PWS. Many of the clinical features described in PWS patients are comparable to those seen in patients with GHD. Studies with growth hormone replacement therapy (GHT), performed mostly in children with PWS but also in adult PWS subjects, show positive effects on height, FM, LBM, visceral adipose tissue, HDL-cholesterol, CRP, adiponectin, physical activity, cardiovascular features, cognition and quality of life without significant impairments in glucose homeostasis [30, 46–55]. Whether the positive effects of GHT differ for PWS patients with and without affirmed GHD is not known.

Further investigations in larger groups of PWS patients are warranted to gain more insight into the exact pathophysiology of the aberrant body composition, appetite regulating hormones, decreased BMD and the metabolic and cardiovascular complications seen in PWS. It is not known whether screening for metabolic and cardiovascular diseases and early preventive treatment with, for instance, statins has a positive effect on morbidity and survival in PWS patients. Also, there are no data on the effects of treatment with calcium and vitamin D or bisphosphonates on BMD and fracture risk. Because of the promising effects of GH therapy thus far, more studies on the long term effects of GHT not only in children with PWS but also in adults are desirable. Furthermore, it is interesting to study whether the effects of GHT are seen in all PWS patients or only in those with affirmed GHD. Whether the hyperphagia and obesity seen in PWS is a result of decreased satiation or increased hunger is still unclear. Therefore, the exact role of appetite regulating factors in individuals with PWS warrants further investigation especially in larger cohorts with older PWS patients. It would be interesting to study changes in body composition and adipokines in course of time, for example before and after weight loss.

Nowadays, there is more awareness of the diagnosis PWS and genetic tests for PWS are more accessible. Thereby, the diagnosis PWS is made at younger age and preventive measures for obesity and treatment with GH can be started early in life. Life expectancy of subjects with Prader-Willi syndrome has increased in recent years and the average BMI, like in our study population, is already lower than reported before. Preventive measures and early treatment can contribute to longer survival, a decrease in morbidity, and possibly a better quality of life and lower healthcare costs.

## Conclusion

In adults with Prader-Willi syndrome alterations in body composition, adipokines, hs-CRP and bone mineral density were demonstrated but these were not associated with IGF-1 levels. Further investigations are warranted to gain more insight into the exact pathophysiology and the role of these alterations in the metabolic and cardiovascular complications seen in PWS. Since there is no cure for PWS and patients with PWS live longer nowadays, prevention and treatment of these complications as early as possible is of great importance.

## Abbreviations

BC: Body composition
BIA: Bioelectric impedance analysis
BMC: Bone mineral content
BMD: Bone mineral density
BMI: Body mass index
DXA: Dual-energy X-ray absorptiometry
FFM: Fat free mass
GH: Growth hormone
GHD: Growth hormone deficiency
GHT: Growth hormone replacement therapy
HOMA-IR: Homeostasis model assessment of insulin resistance
hs-CRP: High-sensitive C-reactive protein
IGF-1: Insulin-like growth factor-1
IGFBP-3: Insulin-like growth factor binding protein-3
LBM: Lean body mass
OGTT: Oral glucose tolerance test
PWS: Prader-Willi syndrome
SDS: Standard deviation scores
WHR: Waist-to-hip ratio
